# Mathematical modeling to predict rice's phenolic and mineral content through multispectral imaging

**DOI:** 10.1016/j.focha.2022.100141

**Published:** 2022-10

**Authors:** Reuben James Buenafe, Rhowell Tiozon, Lesley A. Boyd, Kristel June Sartagoda, Nese Sreenivasulu

**Affiliations:** a Consumer-driven Grain Quality and Nutrition Unit, Rice Breeding and Innovations Cluster, International Rice Research Institute, Los Baños, Philippines; b Max-Planck-Institute of Molecular Plant Physiology, Potsdam-Golm, Germany; c NIAB, 93 Lawrence Weaver Road, Cambridge CB3 0LE, UK

**Keywords:** Pigmented rice, Partial least square regression, Random forest model, Artificial neural network

## Abstract

Over half the world population relies on rice for energy, but being a carbohydrate-based crop, it offers limited nutritional benefits. To achieve nutritional security targets in Asia, we must understand the genetic variation in multi-nutritional properties with therapeutic properties and deploy this knowledge to future rice breeding. High throughput, VideometerLAB spectral imaging data has been effective in estimating total anthocyanin content, particularly bound anthocyanin content, using the high prediction power of partial least square (PLS) regression models. Multi-pronged nutritional properties of phenolic compounds and minerals, together with videometerLAB features, were utilized to develop models to classify a collection of black rice varieties into three distinct nutritional quality ideotypes. These derived models for black rice diversity panels were created utilizing videometerLAB data (L, A, B parameters), selected phenolic types (total phenolics, total anthocyanins, and bound flavonoids), and minerals (Molybdenum and Phosphorous). Random forest and artificial neural network models depicted the multi-nutritional features of black rice with 85.35 and 99.9% accuracy, respectively. These prediction algorithms would help rice breeders strategically breed nutritionally valuable genotypes based on simple, high-through-put videometerLAB readings and a small number of nutritional assays.

## Highlights

• Unraveling the genetic diversity of pigmented rice for multi-nutritional properties.
• Efficient prediction of bioactives through multi-spectral fingerprinting data.
• Random forest and artificial neural network models to predict multi-nutritional traits.
• Linking black rice nutritional value for sustained human health.

## 1 Introduction

Rice is a primary carbohydrate source for over half of the world's population, and global demand is steadily increasing. Consumption in Asia alone, is projected to increase by 67% by 2035 (https://ricepedia.org/challenges/food-security). However, commercial white rice lacks nutritional density and over-reliance on this monocrop diet contributes to the double burden of malnutrition epidemic. With the recent COVID-19 pandemic exacerbating food and nutritional insecurity issues it is important to bring the missing nutrition into popular food crops of cereals by tapping into the genetic wealth potential for multi-nutritional properties (Littlejohn & Finlay, 2021).

Market demand for pigmented rice, distinguished by its red, purple, or black pericarp, has grown due to its dietary and therapeutical traits (Samyor et al., 2017). Pigmented rice in the diet has shown multiple nutritional benefits, including antioxidant, anticancer, antidiabetic, and anti-aging (Mbanjo et al., 2020; Tiozon et al., 2021). These effects have been ascribed to the presence of phenolic compounds such as phenolic acids, flavonoids, and anthocyanins (Callcott et al., 2019). Important phenolic compounds may exist in their free form in the cytoplasm or as conjugates of cell wall polysaccharides and lignin. Moreover, these bioactive compounds in the rice grain are concentrated in the bran layer and husk which contribute to pericarp coloration (Huang & Lai, 2016). In particular, accumulation of anthocyanins gives rice grain its purple/ black color, whereas proanthocyanidins are responsible for the red rice color (Khoo et al., 2017). Consequently, the concentration of bioactive components is higher in pigmented than in non-pigmented rice (Brotman et al., 2021; Goufo & Trindade, 2014). Potential health benefits of pigmented rice and the genetics underlying the accumulation of the bioactive compounds have been reviewed recently (Mbanjo et al., 2020; Tiozon et al., 2021). Pigmented rice also has exceptional genetic diversity for minerals such as iron (Fe), zinc (Zn), magnesium (Mg), copper (Cu), potassium (K) and calcium (Ca) (Jeng et al., 2011; Kang et al., 2011). Higher density of important macroelements (Na, P, S, Ca, K, and Mg) and microelements (Mn, Mo, Zn, Cu, and Fe) have been identified in some rice cultivars. These elements are crucial to human health as they aid in maintaining metabolic and energy homeostasis, proper fluid balance, blood pressure regulation, and overall health of the immune system (Huang et al., 2020). As a result, more consumers have been drawn to health-promoting food products, generating a growing market for more nutritious staples like whole-grain pigmented rice.

Color is an essential feature that consumers consider when selecting various food products. Multispectral phenotyping is a non-destructive and high throughput technique which can be used to measure color changes in fortified rice (Tiozon et al., 2021) and monitor seed quality (ElMasry et al., 2019). Since a relationship between the accumulation of bioactive chemicals in the pericarp and aleurone is related to enriched bioactives, multispectral phenotyping may be deployed to relate the concentration of these phenolic compounds to the color of rice. In our previous work, we have developed models to classify the cooking and eating quality ideotypes of *indica, japonica,* and hybrid rice varieties (Buenafe et al., 2021a, 2021b). To date, however, no model has been developed that utilizes multispectral imaging to predict the nutritional quality of rice by inferring the content of bioactives and minerals. In this study, we relate multispectral imaging data with dietary components such as total phenolics, total flavonoids, total anthocyanins, and micronutrients to predict the nutritional classes of diverse germplasms of pigmented rice and identify rice varieties or accessions with superior dietary composition and health benefits.

## 2 Methods

### 2.1 Rice material

A set (*n* = 385) of diverse germplasm comprising white (*n* = 33) and pigmented rice composed of purple-colored (*n* = 26), variable-purple-colored (*n* = 301), and red-colored (*n* = 25) varieties were selected, planted, and grown during the dry season of 2019 at the International Rice Research Institute (IRRI), Los Baños, Laguna, the Philippines under well-maintained, irrigated and rain-fed conditions. A subset of line (*n* = 200) capturing the diversity of purple and variable purple rice were also planted and grown during the wet season of 2020. Both sets (*n* = 385 and *n* = 200) were planted in a 3-m^2^ effective area plots, excluding the borders, using an augmented randomized complete block experimental design. Both sets followed the same management protocols imposed at IRRI. The grains were harvested and air-dried to 14% moisture content. Then, the grains were dehulled using a rice sheller THU-35A (Satake Corporation, Hiroshima, Japan) and ground to a fine powder (using Mixer Mill MM400, Germany) for biochemical analysis.

### 2.2 Determination of multispectral imaging properties of rice grain using videometerLAB

The multispectral phenotyping of pigmented rice grains was conducted following the protocol described by Mbanjo et al. (2020). Briefly, twenty grains from each accession were placed evenly across a 90 mm Petri dish. The multispectral image of each grain was captured at 19 wavelengths from 365 to 970 nm, each with a resolution of 2056 × 2056 pixels. The color difference metrics defined by the CIE (*Commission International d'Eclairage*) in 1976 and color-appearance attributes, such as a* (green to red shade), b* (blue to yellow shade), L (lightness, clarity of the pericarp), intensity, saturation (i.e., the saturation of color describes its degree of purity in relation to neutral grey) and hue angle (H°, angular specification of the color perceived as red, yellow, blue or green) were measured.

### 2.3 Extraction of free and bound phenolic, flavonoid, and anthocyanin content of rice grains

The free and bound phenolic components of rice grains were extracted according to the method described by Brotman et al. (2021), with minor modifications. Whole grain rice flour (1.0 g) was extracted twice with 10 mL of 80% methanol solution. For each extraction, the mixture was placed in an iced ultrasonic bath for 1 h. Samples were then centrifuged at 12,500 rpm for 20 min. Supernatants from each extraction procedure were pooled and adjusted to pH 4.5–5.0 using 1M HCl or 1M NaOH.

The residues from each centrifugation procedure were mixed with 20 mL of 4 M NaOH for 2 h and placed in an iced ultrasonic bath to release the ester- or ether-linked phenolic compounds. The mixture was centrifuged at 12,500 rpm for 15 min. The supernatant was adjusted to a pH 4.5-5.0 and then, added with 10 mL of ethyl acetate twice to extract the bound phenolics (phenolic acids, flavonoids, and anthocyanins).

### 2.4 Quantification of the nutritional components of rice grain

#### 2.4.1 Estimation of phenolic compounds in rice grain

The total phenolic content (TPC) and total flavonoid content (TFC) were determined using method described in Ti et al. (2014). The total anthocyanin content (TAC) was estimated based on the pH differential method (Lee et al., 2005). Absorbance was measured using a microplate reader (BMG SPECTROstar Nano) at 765 nm for TPC, 510 nm for TFC and 520 and 700 nm for TAC. TAC = A x VM where A = (absorbance at 520 nm - 700 nm) pH 4.5; V = volume of extract (mL) and M = fresh mass of the sample (g). TPC was expressed as mg of gallic acid equivalent (GAE) per 100 g of sample. TFC was calculated using a standard catechin curve and expressed as mg of catechin equivalent (CE) per 100 g of sample. TAC was expressed as cyanidin-3-O-glucoside (Cyn-3-Glu) equivalent. This was done in triplicates.

#### 2.4.2 Mineral content measurement in rice grain

The ground whole grain rice (0.600–0.625 g) were digested using 20 mL of 1% HNO_3_ and subjected to Inductively Coupled Plasma-Optical Emission Spectrometry (ICP-OES) for mineral content determination following the method (Molina et al., 2019). Eleven minerals (Ca, Cu, Fe, K, Mg, Mn, Mo, Na, P, S, and Zn) were quantified.

### 2.5 Multivariate analyses and mathematical modeling

#### 2.5.1 Partial least square regression (PLSR) models for predicting phenolic contents

R (Version 3.3.2, released 2016) was used for all multivariate and statistical analyses. Principal component analysis (PCA) was used to reveal variability in multispectral imaging, phenolic, and micronutrient data.

PLSR is a simple and accurate linear algorithm for modeling spectral data. It is an extensively used approach for predicting the antioxidant content and potential of various plant extracts (Li & Huang, 2021; Sahin & Demir, 2016). To this end, PLSR was applied on the multispectral data of the rice diversity panel (*n* = 385) to predict the TAC, TFC, and TPC values and its free and bound components. The multispectral imaging data was used as inputs to the models to generate predicted outputs, as mentioned previously. The data set (*n* = 385) was divided into 70% training and 30% test sets, using the simple random sampling without replacement method. Ten-fold cross-validation was applied to each model to ensure its stability and validity. The dimensionality of the predictors was reduced using a correlation filter of r ≥ |0.70| and the min-max normalization technique was used as spectra pre-processing tool to ensure that they would have the same contribution to the model. The performance of the developed model was evaluated using the root-mean-square error (RMSE) and coefficient of determination (R^2^).

#### 2.5.2 Nutritional quality classes and classification models for black-colored rice

In general black-colored rice could be distinguished with two unique pericarp hues, purple and variable purple. The purple hue of the pericarp, perceptible to the human eye, distinguishes the rice as being purple. In contrast, the variable purple is marked by the presence of additional colored streaks than purple. Neither multispectral imaging data nor nutritional factors, such as phenolic and micronutrient content, are able to differentiate between these two types of black-colored rice. Thus, reclassification was performed using the nutritional factors to provide consumers with a nutritional rationale for purchasing purple-colored rice. To achieve the new classification, the phenolic and micronutrient content data of all the black rice, which includes the purple- and variable-purple-colored rice (*n* = 327, grown in 2019 dry season) from the same pigmented rice collection, were subjected to Agglomerative nesting via Ward's method (AGNES). The number of best clusters formed from the data set was identified using three internal validation measures (silhouette width, Dunn index, and connectivity) and four stability measures (average proportion of non-overlap, average distance, average distance between means, and figure of merit).

The generated classes were predicted using the colorimetric data to see whether the videometerLAB instrument could be used as a one-stop approach to predict the phenolic content and nutritional value of black rice. Random forest (RF) and artificial neural network (ANN) were utilized as predictive classification models in this study, with the variable inputs consisting of colorimetric data that had been trimmed using the correlation filter. RF models, which have been previously used to predict rice cooking and eating quality classes (Buenafe et al., 2021a), were applied to predict and classify the nutritional quality. RF models used bootstrap to distribute the input variables (*x_i_*) to a distinct class by choosing the majority among all groups of tree-based classifiers (*h*(*x_i_, Θ_k_, k=1)*), where *Θ_k_* are identically and independently distributed random vectors (Tatsumi et al., 2015).

ANN is a machine learning approach which has been shown to be effective in the regression prediction of rice yield (Basir et al., 2021), as well as the prediction of cooking and eating quality classes of hybrid rice (Buenafe et al., 2021b). ANN was applied with a back-propagation algorithm with a three-layer architecture. The input layer comprised of the colorimetric and nutritional data that had been previously trimmed using a correlation filter, whilst the output layer consisted of the classes generated by the cluster analysis. The best number of nodes in the hidden layer was identified through a trial-and-error process. For the final RF and ANN models, 10-fold cross-validation was performed for the whole data set, and its accuracy was calculated based on the percentage of correctly predicted classes.

Both of the models (RF and ANN) were used to predict the nutritional quality classes created through the AGNES clustering. The top five contributing micronutrients and three multispectral imaging parameters (L, A, and B) were used as inputs to the models. Both models were applied to a validation set (*n* = 200) planted during wet season to check its applicability and validity to other conditions.

## 3 Results and discussion

### 3.1 Variation in the multispectral imaging and nutritional components of pigmented rice

**Fig. 1:**
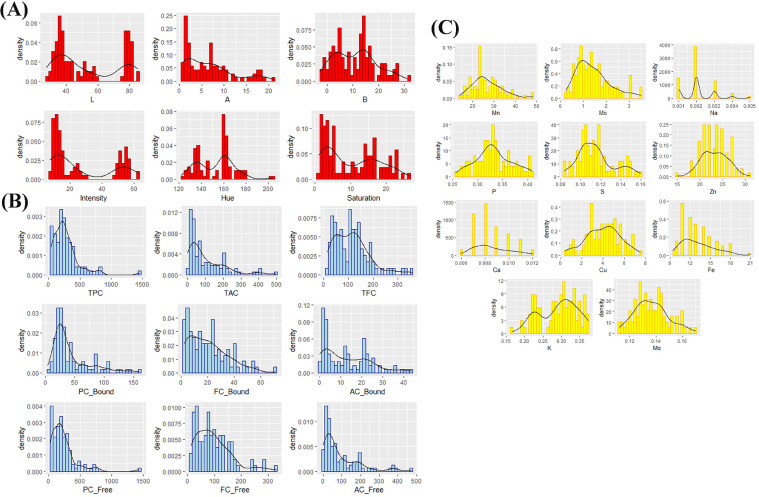
Distribution plots of pigmented rice collection (*n* = 385) based on **(A)** multispectral imaging data obtained from videometer, **(B)** phenolics data and **(C)** minerals data. Abbreviated variables: L- whiteness, A- redness, B- yellowness, TPC – total phenolic content, TFC – total flavonoid content, TAC- total anthocyanin content, PC- phenolic content, FC- flavonoid content, AC- anthocyanin content, Mn - manganese content, Mo- molybdenum content, Na – sodium content, P – phosphorus content, S- sulphur content, Zn- zinc content, Ca- calcium content, Cu- copper content, Fe- iron content, K- potassium content, Mg- magnesium content.

**Fig. 2:**
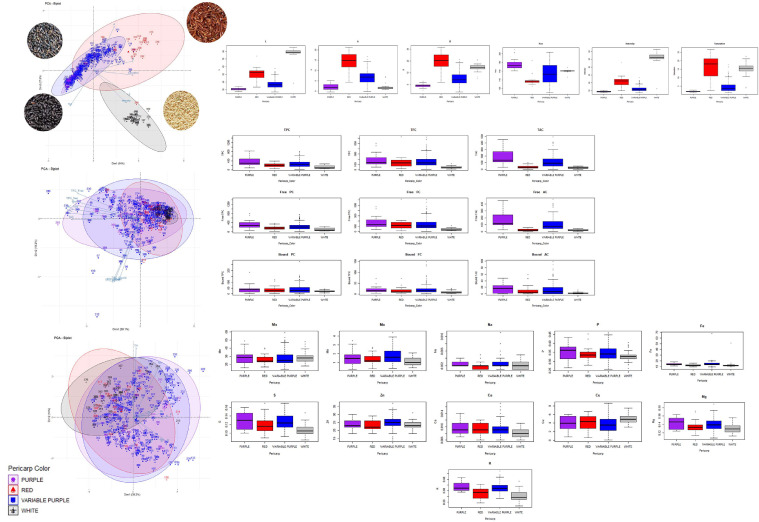
PCA biplots and phenotypic distribution box plots of the pigmented rice collections (*n* = 385) based on multispectral imaging data obtained from videometer, phenolics content and mineral content arranged according to pericarp color (purple, variable purple, red, and white). PCA biplot of colorimetric parameters obtained from videometerLAB showed that 64.0% of variation is explained by PC1 while 27.8% is explained by PC2. PCA biplot of phenolic content of pigmented rice showed that 50.1% of variation is explained by PC1 while 19.2% is explained by PC2. PCA biplot of mineral content of pigmented rice showed that 39.2% of variation is explained by PC1 while 14.0% is explained by PC2. Abbreviated variables: L- whiteness, A- redness, B- yellowness, TPC – total phenolic content, TFC – total flavonoid content, TAC- total anthocyanin content, PC- phenolic content, FC- flavonoid content, AC- anthocyanin content, Mn - manganese content, Mo- molybdenum content, Na – sodium content, P – phosphorus content, S- sulphur content, Zn- zinc content, Ca- calcium content, Cu- copper content, Fe- iron content, K- potassium content, Mg- magnesium content.

**Fig. 3:**
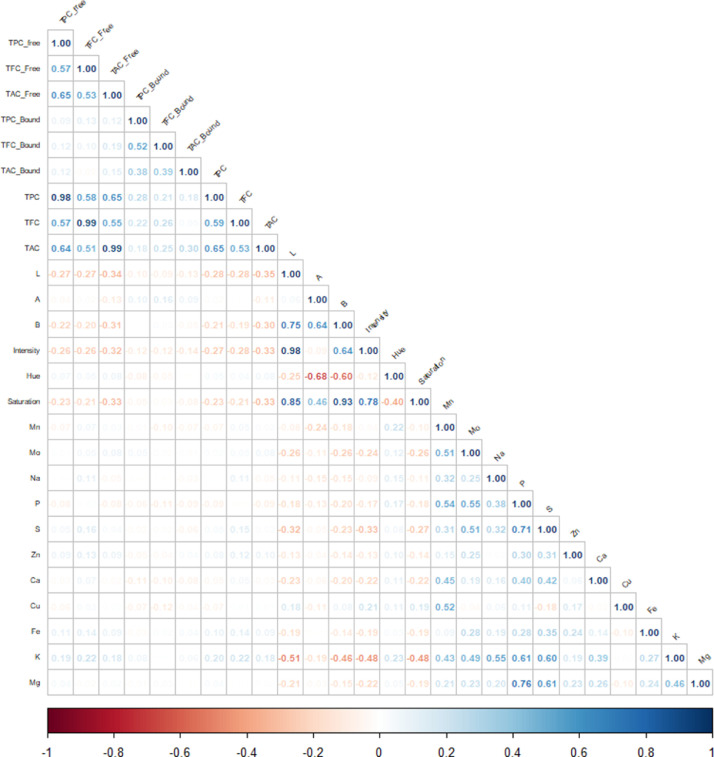
Correlogram of all generated data for the pigmented rice collection (*n* = 385). Abbreviated variables: L- whiteness, A- redness, B- yellowness, TPC – total phenolic content, TFC – total flavonoid content, TAC- total anthocyanin content, PC- phenolic content, FC- flavonoid content, AC- anthocyanin content, Mn - manganese content, Mo- molybdenum content, Na – sodium content, P – phosphorus content, S- sulphur content, Zn- zinc content, Ca- calcium content, Cu- copper content, Fe- iron content, K- potassium content, Mg- magnesium content.

The rice diversity panel (*n* = 385) revealed a wide variation in multispectral imaging properties and nutritional content (Fig. 1). In the present study, L, A, B, intensity, H°, and saturation comprised the multispectral imaging data of rice grain gathered using videometerLAB. The L parameter indicates the whiteness of the sample and ranged from 27.48 to 85.12, with a mean value of 52.74 ± 19.61. The A parameter, which indicates the sample's redness (positive values) and greenness (negative value), ranged from 0.81 to 21.18, with a mean value of 6.59 ± 5.35. The B parameter indicates the yellowness (positive values) and blueness (negative values) of samples and ranged from -2.89 to 31.43, with a mean value of 10.90 ± 8.03. The intensity, H°, and saturation of the samples varied from 7.55 to 62.57, 122.13 to 203.37, and 1.14 to 26.68, respectively, with mean values of 26.87 ± 19.08, 152.78 ± 16.59, and 10.29 ± 7.37. In terms of pericarp coloration (white, red, purple, and variable purple), data distribution showed variability in multispectral imaging properties and nutritional components (Fig. 2). A Kruskall-Wallis test followed by Wilcoxon–Mann–Whitney test for post hoc analysis showed a significant difference (*p* > 0.05) among all rice colors against the L, A, B, intensity, and saturation. For H°, the red and variable purple showed no significant difference from each other. Due to these observed correlations (Fig. 3), the multispectral imaging evidently gives a highly reliable measurement of pericarp coloration, as substantiated by the PCA bi-plots (Fig. 2). PC1 explained 54.9% of the variation, while PC2 accounted for 37.3%. The results showed that most purple and variable, purple-colored rice pericarp had lower L values, intensity, saturation, and B values, whereas white and red pericarps exhibited the opposite. These results can be ascribed to high L values which indicate whiteness of the material and low (negative) B values which indicate the blueness (Vieira et al., 2018). The white and red pericarp colors are very well distinguished by the A and H° parameters, wherein the red pericarp has lower values of H° but higher values of A. This is because the A parameter describes the redness of the material, and the H° is calculated as *tan^−1^(B/A)* (Vieira et al., 2018). Most variable purple-colored rice had higher A and lower H° values than purple. However, there was still a number of variable purple-colored rice with the same multispectral imaging properties as purple-colored rice. Generally, these results shows that videometerLAB parameters could differentiate between black (variable purple and purple), red, and white rice. However, it was unable to distinguish between the purple and variable purple grains. These findings corroborate those reported for Philippine pigmented rice which were discriminated based on multispectral imaging color parameters (Mbanjo et al., 2019).

The phenolic content of the rice collection showed different trends in terms of variability (Fig. 2b). The TPC ranged from 32.31 to 1455.72 mg GAE/100 g DM with a mean value of 268.47 ± 210.33 mg GAE/100 g DM, while the TFC and TAC ranged from 16.06 to 359.43 mg CE/100 g DM and 0 to 494.46 mg Cyn-3-Glu/100 g DM, respectively, with mean values of 113.00 ± 70.67 mg CE/100 g DM and 102.67±104.4 mg Cyn-3-Glu/100 g DM. In general, the colored rice showed greater levels of total phenolics, flavonoids, and anthocyanin than the non-colored rice varieties (Fig. 2b). Among the colored rice, black rice (purple and variable purple) had higher TPC and TAC levels than red rice, as previously shown (Goufo et al., 2014; Shao et al., 2014). Although there was no significant difference among the colored rice in terms of TFC levels, some variable purple rice varieties had lower TFC than white rice. Similar results were observed by Shen et al. (2009). These high TFC in white rice could be due to the presence of flavonoids (e.g., flavones, flavanones, flavonols) other than anthocyanins.

When comparing the free and bound forms of phenolic compounds, the levels of free PC (phenolic content), FC (flavonoid content), and AC (anthocyanin content) were two to three-times higher than the bound form. The free PC, FC, and AC (ranged from 26.98 to 1427.23 mg GAE/100 g DM, 12.47 to 330.09 mg CE/100 g DM, and 0 to 471.82 mg Cyn-3-Glu/100 g DM, respectively, with mean values of 232.65 ± 208.09 mg GAE/100 g DM, 94.00 ± 64.08 09 mg CE/100 g DM, and 90.78 ± 99.20 mg Cyn-3-Glu/100 g DM. The bound forms of PC, FC, and AC ranged from 3.97 to 158.91 mg GAE/100 g DM, 2.29 to 71.06 mg CE/100 g DM, and 0 to 43.6 mg Cyn-3-Glu/100 g DM, respectively, with mean values of 44.75 ± 33.42 mg GAE/100 g DM, 20.65 ± 15.01 mg CE/100 g DM, and 13.29 ± 11.36 mg Cyn-3-Glu/100 g DM. The lower levels of PC, FC, and AC detected in the bound form support the findings of prior studies (de Mira et al., 2009; Sumczynski et al., 2016). However, Shao et al. (2014) reported higher levels of bound phenolics in rice lines. Although phenolic content is primarily influenced by genetics, the quantitative variation may be affected by a range of environmental growth conditions, abiotic and biotic stresses, as well as cultivation techniques (Dey & Bhattacharjee, 2020).

No significant differences were seen in purple and variable purple rice in free or bound phenolic properties. There were also no significant variations in TAC, free AC, bound PC, or bound FC between red and white rice. However, purple rice could be differentiated from red rice by TPC, TAC, free PC, free AC, bound FC, and bound AC. These differences were also observed between red and variable purple rice when comparing the levels of bound PC. Both the purple and variable purple rice showed a significant difference in their TPC, and free and bound phenolic properties when compared with white rice. However, while variation was observed between the levels of these phenolic compounds in different colored rice, the PCA bi-plots indicated that phenolic content could only distinguish between red and white rice. Interestingly, it was observed that the white and red pericarp colors were seen to be dispersed along regions of low TAC, TPC and TFC. Purple and variable purple pericarps, on the other hand, were concentrated along high and low TAC, TPC and TFC regions, although a few values diverged from this trend (Fig. 2b). These findings reveal that although the phenolic content of rice could not make a distinction between the four pericarp colors, it could distinctly distinguish black rice from other pericarp colors. Based on the specific phenolic acids, anthocyanins, and proanthocyanins, Shao et al. (2018) were able to discriminate between brown, red, and black rice. However, Zhang et al. (2008) demonstrated that near-infrared spectroscopy models could not determine the TFC based on pericarp colors alone. In addition, the total metabolite content, based on spectrophotometric methods (TPC, TFC, and TAC), failed to fully distinguish between rice types (Zhang et al., 2008). As phenolic content varies widely across colored rice samples (Mbanjo et al., 2020), it appears that identifying particular phenolic compounds would be a more efficient method of grouping rice samples using PCA than utilizing generic phenolic content.

High correlations between the nutritional and spectral properties were observed in the rice collection (Fig. 3). TPC, TFC, and TAC were shown to have a very strong positive association with their free equivalents, while their bound counterparts all showed negligible correlations. Since the concentrations in free form are higher than in bound, the levels of free PC, FC, and AC, respectively, had a considerable impact on the quantity of TPC, TFC, and TAC, while the levels of bound PC, FC, and AC had less of an impact. TFC and TAC are moderately correlated with TPC, indicating that varieties which accumulate higher flavonoid and anthocyanins also develop greater levels of other phenolic compounds (Muflihah et al., 2021; Shen et al., 2009).

Six major minerals (Na, P, S, Ca, K, and Mg) and five trace elements (Mn, Mo, Zn, Cu, and Fe) were quantified across the rice collection (Fig. 1). K and P were the most abundant elements, each accounting for almost 40% of the total mineral content. The macro elements Na, P, S, Ca, K, Mg, had the following value ranges: 0 to 0.01%, 0.26 to 0.41%, 0.08 to 0.16%, 0.44 to 7.5%, 0.17 to 0.37%, 0.11 to 0.17%, while micronutrients Mn, Mo, Zn, Cu, and Fe exhibit the range of 14 to 48 mg/kg, 0.23 to 3.5 mg/kg, 15 to 3 mg/kg, 0.009 to 0.01 mg/kg, and 9.4 to 21 mg/kg.

Significant differences were observed between red and purple rice in terms of Na, S, Fe, K, and Mg content, and between red and variable purple rice for Na, S, Fe, K, Mg, Mo, and Cu (Fig. 2c). S, Ca, Fe, K, and Mg content between white and purple rice showed significant variations, as did S, Fe, K, and Mg between white and variable purple rice. No significant differences were observed between purple and variable purple, and between red and white rice, in terms of mineral composition. Only P and S, and P and Mg showed a strong positive correlation between minerals (Fig. 3). Mg, P, and S are vital macronutrients required for plant functions such as enzyme activation/deactivation, energy generation, nitrogen fixation, carbohydrate and nucleic acid metabolism, photosynthesis, redox reactions, and signaling mechanisms (Hörtensteiner, 2009). Hence, the strong correlation between Mg, P, and S in rice may be due in part to their interactions in these activities. As with phenolic content, the mineral content does not completely discriminate the rice lines with respect to coloration (Fig. 2). Pigmented rice genotypes with greater Fe and Zn concentrations than non-pigmented rice have been reported (Tiozon et al., 2021). While our data indicated that the concentration of Fe varied by rice color, the concentrations of micronutrients Zn and Mn did not differ substantially. In general, pigmented rice has been found to have higher total mineral content than white rice (Goufo & Trindade, 2014). However, rice exhibits considerable ionomic variation (Pinson et al., 2014), and unlike anthocyanins and proanthocyanidins, the mineral content does not contribute to pericarp color (Shao et al., 2018). All these factors contribute to the improbability of discriminating the mineral content of whole grain rice based on grain color alone.

Although the multispectral imaging variables have shown stronger correlations to distinguish the purple, variable purple, red and white rice diversity collection, these multi-spectral parameters did not demonstrate correlations with the nutritional components. For instance, L exhibited a strong positive relationship with A, B, and saturation. Likewise, videometerLAB parameters A and B demonstrated strong, positive relationships with saturation, and strong negative relationships with H°. A strong correlation was also observed between A and B, and between B and intensity. The broad range of nutritional component concentrations and spectral features indicate that the collection of rice samples selected for mathematical modeling is robust.

### 3.2 PLSR model of multispectral imaging to predict bioactive compounds in the pigmented rice

On the basis that both the multispectral imaging parameters and phenolic content could partially distinguish between the black rice (purple and variable purple) and the other pericarp colors based on PCA, it was hypothesized that there could be a direct relationship between the two sets of parameters in rice. Various studies have used colorimetric parameters to predict anthocyanin and total phenolic content of a variety of fruits and vegetables (Vieira et al., 2018). To the best of our knowledge, this approach has not been applied to colored rice. We, therefore, applied the PLS regression model to predict TAC, TPC, and TFC and its free and bound components in rice based on multispectral imaging parameters.

In order to preclude overfitting issues, dimensionality reduction was performed using a correlation filter prior to model calibration. It was found that only L, A, B, and H° were suitable variables to be used as input. Both saturation and intensity are derived quantities from A and B, and their high and positive correlations render them unsuitable. The R^2^ and RMSE values for both the training and test sets are shown in Table 1. Based on both the R^2^ and RMSE values, the models produced favorable outcomes. The models demonstrated that the TPC, TFC, and TAC could be predicted using colorimetric parameters with high accuracy, indicating that the videometerLAB could be used as a fast and effective one-stop solution for estimating TPC, TFC, and TAC in colored rice (Table 1). Furthermore, bound AC, PC, and FC showed better R^2^ and RMSE values compared to their free counterparts. Even though free AC, PC, and FC are more highly correlated with TAC, TPC, and TFC, the multispectral imaging more accurately predicts the levels of bound AC, PC, and FC, rather than the free fractions. Although the results of the models are optimum, these must be used in caution since environmental factors and growing conditions might affect the values of phenolics, flavonoids and anthocyanins in plants.

**Table 1:** Results of partial least-square (PLS) models from multi-spectral imaging data to predict free, bound, and total phenolic content of black (variable purple and purple), red, and white rice diverse lines (*n* = 385)

Predicted Variables	Training (*n* = 270)		Testing (*n* = 115)	
	R^2^	RMSE	R^2^	RMSE
Free Phenolic Content	0.66	0.18	0.73	0.22
Free Flavonoid Content	0.62	0.25	0.51	0.40
Free Anthocyanin Content	0.36	0.58	0.28	0.89
Bound Phenolic Content	0.61	0.55	0.63	0.42
Bound Flavonoid Content	0.73	0.41	0.75	0.51
Bound Anthocyanin Content	0.82	0.32	0.83	0.28
Total Phenolic Content	0.71	0.60	0.79	0.63
Total Flavonoid Content	0.79	0.52	0.80	0.61
Total Anthocyanin Content	0.80	0.51	0.84	0.69

Bound phenolics are covalently bound to cell wall structural components, e.g., cellulose, hemicellulose, lignin and pectin (Acosta-Estrada et al., 2014). This model implies that the phenolics trapped in the cellulosic materials of the rice bran can greatly influence the surface grain color of rice. Since TAC, TPC, and TFC are the sum of the free and bound components, it can be inferred that high levels of free components will contribute to lower accuracies of TAC, TPC, and TFC estimations using multispectral imaging data. To further establish the association between bound phenolics and the pigmentation of rice, future modeling efforts might include the degree of milling as a variable. Our findings also reveal that the models have higher prediction accuracy for TAC, followed by TFC, and TPC. Although TPC and TFC may contribute to the pigmentation, anthocyanins have a significant relationship with the dark pigmentation of black rice. Anthocyanin accumulates during the grain developmental stage, reaching its peak at rice maturation (Mackon et al., 2021). About 97% of TAC in black rice seed concentrated in the bran, including the pericarp, aleurone layer and seed coat, while trace amounts are found in the endosperm. Pericarp color can be used to predict TAC due to the ubiquitous presence of the auxochromic group in anthocyanins. The basic chromophore of anthocyanins is the 7-hydroxyflavilyum ion. This chromophore has eight conjugated double bonds which possess a positive charge on the heterocyclic oxygen ring resulting in an intense red-orange to blue-violet color under acidic solutions (Bueno et al., 2012).

### 3.3 Nutritional quality classes and classification models for black-colored rice

**Fig. 4:**
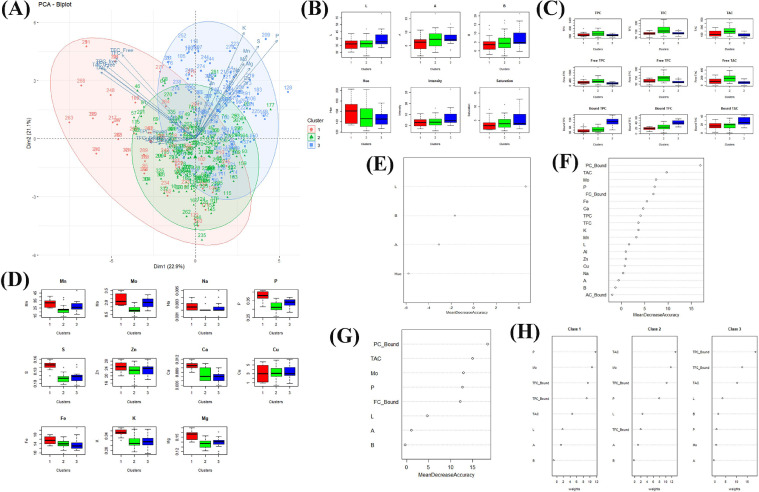
Nutritional quality biplot and RF model variable importance **(a)** PCA biplot of the three nutritional quality classes based on phenolic and micronutrient contents wherein PC1 explains 22.6% of the variation while PC2 explains 14.4% of the variation. Phenotypic distribution box plots of the pigmented rice collections based on **(b)** VideometerLAB parameters **(c)** phenolics content and **(d)** mineral content arranged according to cluster (1,2 and 3). Resulting variable importance of the random forest models generated using **(f)** multi-spectral imaging parameters, **(e)** the combinations of multi-spectral imaging, phenolic content and mineral content data **(g)** the top 5 important variable and multi-spectral imaging data, and **(h)** individual variable contributions per class. Abbreviated variables: L- whiteness, A- redness, B- yellowness, TPC – total phenolic content, TFC – total flavonoid content, TAC- total anthocyanin content, PC- phenolic content, FC- flavonoid content, AC- anthocyanin content, Mn - manganese content, Mo- molybdenum content, Na – sodium content, P – phosphorus content, S- sulphur content, Zn- zinc content, Ca- calcium content, Cu- copper content, Fe- iron content, K- potassium content, Mg- magnesium content.

After demonstrating that the multispectral parameters captured by the videometerLAB could be used to estimate the phenolic content of black rice, we sought out to determine the potential of multispectral imaging to predict the overall nutritional value of black rice. Also we noted higher genetic variability for multi-nutritional properties (minerals and bioactives) within the purple and variable rice's (Fig. 2) hence we considered black rice collection for modeling to predict nutritional quality of multiple nutrients. The nutritional quality of rice is a multidimensional trait that is heavily influenced by the biochemical composition (Mbanjo et al., 2020). In this study, only phenolic and micronutrient content of rice were chosen to identify nutritional quality classes, as they are responsible for numerous health benefits, including anti-aging, anti-inflammatory, antioxidant and antiproliferative activities (Lin et al., 2016). In addition, the identified quality classes would only describe those of black rice, since the PCA results, based on videometerLAB properties (Fig. 2a), showed distinctness among red and white rice when compared using phenolic and micronutrient content. Using the parameters derived from the process of dimensionality reduction, the cluster analysis uncovered three distinct quality classes (Fig. 4). Class 1 comprised of black-colored rice with high micronutrient content, low total phenolics (TAC, TPC and TFC) and low bound phenolics. Class 2 were rice varieties with low levels of micronutrients, high TPC, TAC, and TFC, and low bound PC, AC, and FC. Class 3 consisted of rice varieties that had high levels of bound AC, FC, and PC, with moderate levels of micronutrients. The PCA biplot derived from phenolic and micronutrient values also demonstrated class distinctions (Fig. 4).

Models using RF and ANN were first created to predict the nutrient classes by deploying only the multispectral measurements produced by the videometerLAB using 2019 dry season black rice collection. These models showed low accuracies of 29.41% and 35.29% for RF and ANN, respectively, indicating that the multispectral parameters were unable to accurately characterize the nutritional quality. However, the RF model showed that among the L, A, B, and H^o^ parameters, the L parameter was the most crucial variable as it contributes most to the model accuracy (Fig. 4). To increase model accuracy, we included the micronutrient and phenolics data as inputs in addition to the multispectral parameters. Dimensionality reduction by correlation filter was performed to avoid overfitting, lowering the number of input variables to 19 (TPC, TFC, TAC, bound PC, bound FC, bound AC, L, A, B, Mn, Mo, Na, P, Zn, Al, Ca, Cu, Fe, and K). All of these properties boosted the accuracy of the RF and ANN models to 78.47% and 85.3%, respectively (Table 2), with the optimal architecture for the neural network containing 23 nodes in the hidden layer.

**Table 2:** Results of random forest and artificial neural network classification models to predict the multi-nutritional classes of black (variable purple and purple) rice. Abbreviated variables: L- whiteness, A- redness, B- yellowness, TAC – total anthocyanin content, TFC – total flavonoid content, TPC – total phenolic content, FC – flavonoid content, PC – phenolic content, AC – anthocyanin content.

Model	Predictors	Number of Nodes in Hidden Layer	Model Accuracy
Artificial Neural Network (2019 Dry Season, *n* = 327)	L, A, B, Hue, Intensity	8	36.42%
	Bound PC, TAC, Mo, P, Bound FC, Fe, Ca, TPC, TFC, K, Mn, L, Al, Zn, Cu, Na. A. B. Bound AC	23	85.35%
	Bound PC, TAC, Mo, P, Bound FC, L, A, B	9	99.9%
Random Forest (2019 Dry Season, *n* = 327)	L, A, B, Hue, Intensity	Not Applicable	30.21%
	Bound PC, TAC, Mo, P, Bound FC, Fe, Ca, TPC, TFC, K, Mn, L, Al, Zn, Cu, Na. A. B. Bound AC	Not Applicable	78.47%
	Bound PC, TAC, Mo, P, Bound FC, L, A, B	Not Applicable	85.3%
Artificial Neural Network (2020 Wet Season Validation Set, *n* = 200)	Bound PC, TAC, Mo, P, Bound FC, L, A, B	9	87.6%
Random Forest (2020 Wet Season Validation Set, *n* = 200)	Bound PC, TAC, Mo, P, Bound FC, L, A, B	Not Applicable	75.43%

Although high levels of accuracy were obtained, it is vital to determine the minimum number of parameters needed to predict the nutritional value of a rice variety. This minimizes the number of biochemical tests and maximizes experimental throughput, hence reducing the cost of chemicals and other resources. A total of eight variables were used to derive the models: bound PC, TAC, and bound FC, Mo, and P levels and L, A, and B parameters from the videometerLAB data. These variables were chosen based on their contributions to the accuracy of the RF model developed when 19 variables were used as inputs. These eight variables were subsequently used to generate the RF and ANN models, which had accuracies of 85.35% and 99.9%, respectively. Evidently, ANN is a stronger model than RF for predicting the nutritional quality of black rice. Among these variables, the bound PC was a major contributor to the accuracy of the RF model (Fig. 4). Moreover, the importance of the variables to the prediction of each nutritional class differed. For Class 1, P was the most important variable due to the abundance of micronutrients within this class. For Class 2, TAC was the most relevant variable, as this class had high total phenolics. Lastly, for Class 3, bound PC was the most important variable as the rice samples within this group have distinctly high levels of bound phenolic compounds.

The applicability and validity of the created models were also tested for reproducibility between independent seasons (dry season versus wet season). Data from black rice collection planted during wet season was used and a result of 87.6% and 75.43% accuracy for both ANN and RF models, respectively, were obtained (Table 2). This shows that even when planted in different seasons, the models will still render accurate results.

## 4 Conclusion

Multispectral imaging is a non-destructive and fast phenotyping technique that provides data on a number of spectral, color-related parameters. Using this technique, black (purple and variable purple) rice may be distinguished from other differently colored rice, i.e., red, and white. This work is the first to construct models that correlate the nutritional components of rice with its pericarp color. The PLS models which utilized videometerLAB data, accurately predicted TAC values based on pericarp color, but TPC and TFC values were predicted with moderate accuracy. Comparing bound and free components, bound PC, FC, and AC produced higher accuracies, implying the importance of bound phenolics in rice bran pigmentation. In summary, it was observed that polyphenols contribute to rice coloration and that these phenolic compounds can be predicted using multispectral imaging parameters. Although color alone is insufficient to identify all nutritional characteristics in rice, our mathematical models could deduce that black rice is superior in phenolic and micronutrient content. When selected phenolic compounds and minerals were used in conjunction with videometerLAB data, the derived ANN and RF models were able to predict the phenotypic variability of multi-nutritional properties within the black rice core collection. Here, we determined that ANN models were comparatively more accurate at predicting the nutrient classes than the RF models. ANN models facilitate the generalization of input-output linkages and the inference of associations on unseen data. The models generated in this work yielded three key nutritional classes for black rice based on its micronutrient and phenolic content. Taken together, these models can be useful for rice breeders as it may enable them to predict the phenolic and mineral content of rice based on its color and a few biochemical indicators. The use of the models created in this study is straight-forward. All the key predictors must be generated in the lab and data results should be fed to the models to predict nutritional quality classes. With this information, breeders can strategically enhance the nutritional status of rice for consumers.

## CRediT authorship contribution statement

**Reuben James Buenafe:** Methodology, Investigation, Data curation, Writing – original draft. **Rhowell Tiozon:** Methodology, Investigation, Writing – original draft. **Lesley A. Boyd:** Writing – review & editing. **Kristel June Sartagoda:** Writing – review & editing. **Nese Sreenivasulu:** Supervision, Investigation, Writing – review & editing.

## Declaration of Competing Interest

We declare no conflict of interest regarding this manuscript.
